# S3-Leitlinie Depressive Störungen im Kindes- und Jugendalter: Wo geht es hin?

**DOI:** 10.1007/s00103-023-03721-4

**Published:** 2023-05-25

**Authors:** Gerd Schulte-Körne, Cosima Klingele, Carolin Zsigo, Maria Kloek

**Affiliations:** Klinik und Poliklinik für Kinder- und Jugendpsychiatrie, Psychosomatik und Psychotherapie, LMU Klinikum, Nußbaumstr. 5a, 80336 München, Deutschland

**Keywords:** Leitlinie, Depression, Kinder, Jugendliche, Behandlung, Guideline, Depression, Children, Adolescents, Treatment

## Abstract

Im Juli 2013 wurde die erste deutschsprachige Leitlinie zur Behandlung depressiver Störungen bei Kindern und Jugendlichen veröffentlicht. Aktuell befindet sich die Leitlinie in einem Revisionsprozess, in welchem die Empfehlungen erneut betrachtet und aktualisiert werden. In diesem Bericht sollen ein Überblick über den aktuellen Stand sowie ein Ausblick auf die zukünftige Entwicklung der Revision gegeben werden.

Innerhalb des Revisionsprozesses wurden die Schlüsselfragen der Erstfassung durch neue Schlüsselfragen erweitert, welche unter anderem den Bereich der ergänzenden Therapien, also Therapien, die zusätzlich zur üblichen Behandlung eingesetzt werden können, sowie den Übergangsbereich vom Jugend- ins Erwachsenenalter abdecken. Zu allen Schlüsselfragen wurden aktualisierende systematische Literaturrecherchen durchgeführt, wobei sowohl randomisierte kontrollierte Studien als auch systematische Übersichtsarbeiten und nicht-kontrollierte Interventionsstudien berücksichtigt wurden. Die Studien wurden daraufhin auf Anwendbarkeit und mögliche Biasrisiken geprüft, sodass Evidenzgrade vergeben werden konnten, welche die Qualität und Relevanz der verfügbaren Evidenz widerspiegeln.

In diesem Bericht wird ein kurzer Überblick über die wichtigsten Erkenntnisse, die aus der neuen Evidenzlage geschlossen werden können, gegeben. Während sich im Bereich Psychotherapie keine wesentlichen neuen Erkenntnisse ergaben, hat sich die Evidenzlage zu einigen Antidepressiva geändert. Im Bereich der ergänzenden Therapien wurde vor allem neue Evidenz zu sportlicher Aktivität gefunden. Allgemein ist mit Änderungen an den Empfehlungen zu Erst- und Alternativbehandlungen zu rechnen. Ein Abschluss des Revisionsprozesses und die Veröffentlichung der revidierten Leitlinie sind bis Ende 2023 geplant.

## Abstract (en)

The German guideline for the treatment of depressive disorders in children and adolescents was first published in July 2013. Currently, this guideline is undergoing revision, in which the recommendations of the original version of the guideline are revisited and updated. This report aims to give an overview of the current status and the next steps for this revision.

As part of the revision process, the key questions posited in the original version were expanded upon. In this, new questions were added regarding complementary therapies, that is, therapies meant to be administered in addition to the usual treatment, as well as regarding the transitionary period from adolescence into adulthood. For all key questions, new systematic literature searches were conducted in order to update the relevant evidence. For this, randomized controlled studies, systematic reviews, and non-controlled intervention studies were included and rated according to their relevance as well as possible risks of bias. Thus, all studies could be assigned a level of evidence that takes into account both the quality and the importance of the study to the guideline.

This report will give a brief overview of the most important insights resulting from the new evidence base identified for the revision. While insights regarding psychotherapy are largely unchanged, there are changes in the evidence for certain antidepressants. In the field of complementary therapies, new evidence has been found for physical activity. In general, it is likely that most recommendations of the original guideline regarding first- and second-line treatments will be updated. The completion of the revision and the publishing of the revised guideline are expected to take until the end of 2023.

## Einleitung

Im Juli 2013 wurde die erste deutschsprachige Leitlinie zur Behandlung depressiver Störungen bei Kindern und Jugendlichen publiziert. Der Anlass dazu war, dass die zu dem Zeitpunkt bereits veröffentlichte Nationale Versorgungsleitlinie „Unipolare Depression“ die evidenzbasierte Forschung zu Kindern und Jugendlichen nicht berücksichtigt hat und damit die notwendigen alters- und entwicklungsspezifischen Behandlungsempfehlungen nicht enthalten waren. Die Leitlinie wurde von den Fachverbänden sehr begrüßt und die Empfehlungen wurden auf Kongressen und Fortbildungsveranstaltungen verbreitet und diskutiert.

Seit der Veröffentlichung wurde eine Studie zur Wirksamkeit der Leitlinie durchgeführt [1]. Anhand von Versicherungsdaten der Barmer Krankenversicherung wurde die Verschreibungspraxis von Antidepressiva bei Kindern und Jugendlichen mit einer depressiven Störung vor und nach dem Erscheinen der Leitlinie untersucht. Es zeigte sich eine Zunahme der Verordnung von Antidepressiva, die in der Leitlinie empfohlen werden (Fluoxetin), und eine Abnahme der darin nicht empfohlenen Antidepressiva (trizyklische AD). Um die Verbreitung und die Akzeptanz der Inhalte der Leitlinie für die Zielgruppe der Kinder und Jugendlichen zu fördern, ist eine kinder- und jugendgerechte Internetseite entstanden (www.ich-bin-alles.de), die sowohl hinsichtlich des sprachlichen Ausdrucks als auch der Bildsprache Kinder und Jugendliche altersgemäß über Depression (Symptomatik, Ursachen, Verlauf) sowie die Leitlinienempfehlungen zur Behandlung informiert [2].

Nach 5 Jahren verlieren Leitlinien ihre Gültigkeit und ein Revisionsprozess wird eingeleitet.

Dieser Bericht beschreibt das Vorgehen bei der Überarbeitung der S3-Leitlinie zur Behandlung depressiver Störungen bei Kindern und Jugendlichen und wesentliche Ergebnisse der Literaturrecherche. Da die neuen Leitlinienempfehlungen noch nicht diskutiert und konsentiert sind, können die abschließenden Empfehlungen an dieser Stelle nicht genannt werden. Dieser Prozess wird bis Ende 2023 abgeschlossen sein.

## Die Revision der S3-Leitlinie

### Vorgehen, Schlüsselfragen und Zielgruppe

Für den Revisionsprozess der S3-Leitlinie wurde wieder ein Steuerungsgremium einberufen, das sich aus Vertreter*innen der folgenden Fachgesellschaften bzw. Verbände zusammensetzt: Deutsche Gesellschaft für Kinder- und Jugendpsychiatrie, Psychosomatik und Psychotherapie e. V. (DGKJP), Berufsverband für Kinder- und Jugendpsychiatrie, Psychosomatik und Psychotherapie in Deutschland e. V. (BKJPP), Bundesarbeitsgemeinschaft der Leitenden Klinikärzte für Kinder- und Jugendpsychiatrie, Psychosomatik und Psychotherapie e. V. (BAG KJPP), Bundespsychotherapeutenkammer (BPtK). Das Steuerungsgremium hat auf der Grundlage der bestehenden S3-Leitlinie, der klinischen Praxis und der neueren Evidenz aus Therapiestudien die Schlüsselfragen für die Literaturrecherche überarbeitet und neue Fragen aufgenommen. Die Schlüsselfragen (zusätzliche neue Schlüsselfragen in kursiv) sind:

1. Welche Rahmenbedingungen für eine Behandlung sollen geschaffen werden?
2. Welches Behandlungssetting (ambulant, teilstationär, vollstationär) kann bei welchen Indikatoren empfohlen werden?
3. Therapie der ersten Wahl:
  1. Was ist die Therapie der ersten Wahl bei Kindern und bei Jugendlichen mit verschiedenen Schweregraden depressiver Störungen?
  2. Welche Therapie der ersten Wahl kann bei rezidivierenden depressiven Störungen eingesetzt werden, um Rückfällen und Wiederauftreten vorzubeugen?
4. *Welche ergänzenden Therapien sind bei Kindern und bei Jugendlichen mit depressiven Störungen empfehlenswert beziehungsweise nicht empfehlenswert?*
5. Welche Therapien sind bei Kindern und Jugendlichen mit depressiven Störungen nicht empfehlenswert?
6. Wann und wie wird der Therapieeffekt festgestellt?
7. Falls eine Therapie nicht wirksam ist, welche Therapie ist als Alternative empfehlenswert?
8. Wie lange wird die Behandlung fortgesetzt und wie wird sie beendet?
9. *Welche spezifischen Therapien und Versorgungssettings sind für Heranwachsende im Übergang vom Jugendalter ins Erwachsenenalter empfehlenswert?*

Die Schlüsselfrage 9 zu den Heranwachsenden ist aufgrund der Schaffung einer neuen, dringend notwendigen Versorgungsstruktur der teil- und vollstationären Behandlung von jugendlichen Patient*innen im Alter von 16–24 Jahren auf sogenannten Transitionsstationen entstanden. Damit wurde eine Versorgungslücke beim Übergang von der kinder- und jugendpsychiatrischen Versorgung in die erwachsenenpsychiatrische Versorgung geschlossen. Die Leitlinie soll überprüfen, ob es für diese Altersgruppe Evidenz für spezifische Behandlungssettings und -methoden gibt und diese ggf. in einer Behandlungsempfehlung abbilden.

Eine weitere Neuerung bei der Revision der Leitlinie ist die Beteiligung von Jugendlichen mit einer Depression im Alter von 13–16 Jahren im Rahmen einer Fokusgruppe. Das Ziel der Fokusgruppe war es, zu erfahren, welche Therapieeffekte und welche Aspekte der Behandlung die Jugendlichen besonders wichtig finden. Zu den wichtigen Therapieeffekten gehören die Verringerung der Suizidalität, die Reduktion der depressiven Stimmung und die Steigerung der Fähigkeiten zur Bewältigung von Alltagsaufgaben, wie zum Beispiel der Schulbesuch. Aus Sicht der Jugendlichen sind die Partizipation bei Therapieentscheidungen und die Beteiligung der Familie an der Therapie zentrale Aspekte.

Die Zielgruppe der Leitlinie sind Kinder und Jugendliche im Alter von 3 bis 18 Jahren mit depressiven Störungen gemäß der Internationalen statistische Klassifikation der Krankheiten und verwandter Gesundheitsprobleme, 10. Revision (ICD-10), d. h. mit

- depressiven Episoden (F32.0/F32.1/F32.2/F32.8/F32.9),
- einer schweren depressiven Episode mit psychotischen Symptomen (F32.3),
- rezidivierenden depressiven Störungen (F33.0/F33.1/F33.2/F33.4/F33.8/F33.9),
- einer rezidivierenden depressiven Störung, gegenwärtig schwere Episode mit psychotischen Symptomen (F33.3),
- Dysthymia (F34.1) und
- Störungen des Sozialverhaltens mit depressiver Störung (F92.0).

Zusätzlich gehören zur Zielgruppe Kinder und Jugendliche, bei denen über die genannten Störungen hinaus noch weitere psychische oder somatische Erkrankungen (Komorbiditäten) vorliegen.

Die Basis für die Leitlinienempfehlungen ist die Bewertung von randomisierten kontrollierten Interventionsstudien, systematischen Reviews und Metaanalysen. Dazu wurden für die Revision wie bereits zuvor Literaturrecherchen zu den folgenden Themenblöcken durchgeführt: Behandlungssetting, Psychotherapie, Pharmakotherapie, Kombinationsbehandlung aus Psycho- und Pharmakotherapie, ergänzende Therapien und Übergangsbereich (Studien zur Behandlung in der Transition vom Jugend- ins Erwachsenenalter). Der Suchzeitraum für die Literaturrecherche erstreckte sich vom Ende der letzten Recherche für die Erstfassung der Leitlinie (Januar 2012) bis zum Juli 2021. Die Ergebnisse werden in 4 Evidenztabellen zusammengefasst, die mit der Leitlinie publiziert werden. Die Methodik der Leitlinie ist in einem Methodenreport genau beschrieben.

**Figure 1:**
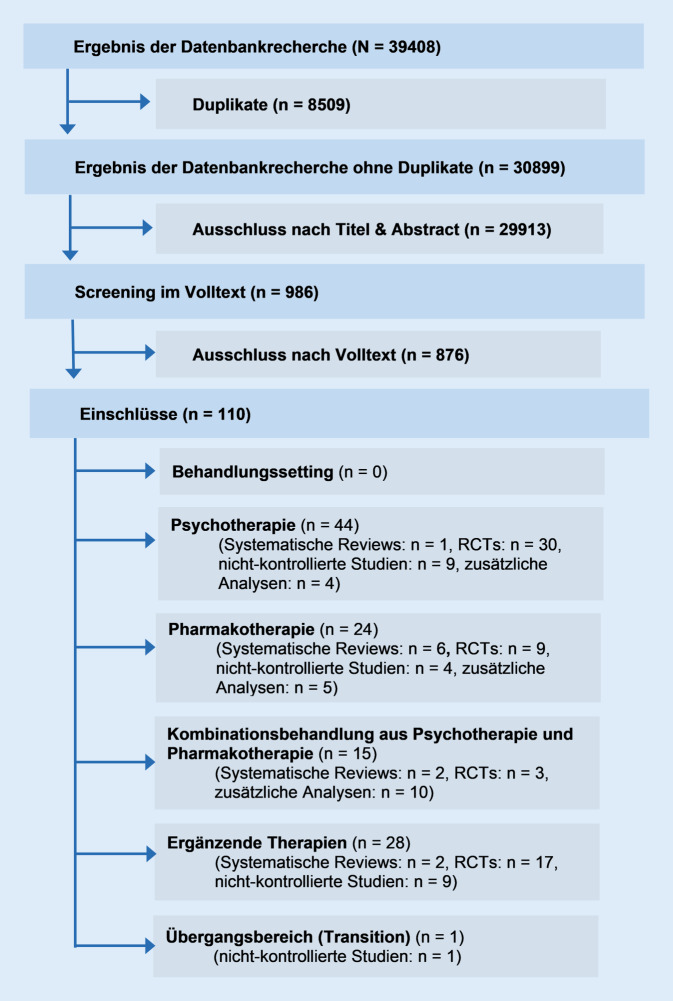

Abb. 1 gibt eine Übersicht, wie viele Studien zu den einzelnen Themenbereichen eingeschlossen werden konnten.

### Bewertung der Evidenz

Ein zentrales methodisches Instrument zur Bewertung der Studien ist das *Bewertungsinstrument Cochrane Risk of Bias Tool 2* (Cochrane RoB‑2, [3]), welches für die randomisierten kontrollierten Studien (RCTs) eingesetzt wurde. Das Cochrane RoB‑2 umfasst 5 Komponenten (Domänen): (1) Biasrisiko aufgrund des Randomisierungsprozesses, (2) Biasrisiko aufgrund von Abweichungen der beabsichtigten Interventionen, (3) Biasrisiko aufgrund der fehlenden Daten, (4) Biasrisiko bei der Outcome-Erhebung und (5) Biasrisiko bei der Auswahl des gemeldeten Outcomes. Jede einzelne Domäne wird entweder mit einem geringen, mäßigen oder hohen Biasrisiko bewertet. Am Ende ergibt sich eine Gesamteinschätzung des Biasrisikos als gering, mäßig oder hoch.

Nach Abschluss der Bewertung wird für die Studien ein Evidenzgrad zwischen 1 (hoch) und 5 (sehr schwach) bestimmt. Dieser beruht auf der Beurteilung des Biasrisikos (s. oben), der Anwendbarkeit der Studie (d. h., wie gut die Studie zu den Fragestellungen der Leitlinie passt), der Genauigkeit der Ergebnisse (d. h., ob eine gute Teststärke oder große Stichprobe vorliegt) sowie der Effektstärke der Ergebnisse.

Einen weiteren wichtigen Bestandteil der Evidenz bilden die systematischen Reviews, d. h. Übersichtsarbeiten von hoher methodischer Qualität, die mehrere RCTs umfassen. Diese Reviews wurden mit dem Bewertungsinstrument „Risk of Bias in Systematic Reviews“ (ROBIS, [4]) methodisch geprüft. Hierbei werden 4 Domänen berücksichtigt: (1) Biasrisiko bei den Einschlusskriterien der Studien, (2) Biasrisiko bei der Identifikation und Auswahl der Studien, (3) Biasrisiko bei der Datenextraktion und Bewertung der Studien, (4) Biasrisiko bei der Datensynthese und den Ergebnissen. Auch hier wird am Ende ein hohes, mäßiges oder geringes Biasrisiko vergeben. Bei systematischen Reviews wird nur die Methodik der Arbeit beurteilt, ohne eine Aussage über die enthaltenen Primärstudien zu treffen. Bei einer guten Methodik des Reviews werden die Einschätzungen der Autor*innen hinsichtlich der zugrunde liegenden Studien übernommen, bei schlechter Methodik werden die Einschätzungen der Autor*innen kritisch reflektiert.

Nicht-randomisierte kontrollierte Studien und nicht-vergleichende Studien stellen den kleinsten Teil der Evidenz dar, die in der Revision der Leitlinie berücksichtigt wurde. Sie wurden nicht mithilfe von Bewertungsinstrumenten geprüft; stattdessen wurde hier nur ein Evidenzgrad festgelegt, welcher auf Basis der vergleichsweise schlechteren Methodik der Studien automatisch niedrig angesetzt wird.

## Neue Erkenntnisse: Wesentliche Ergebnisse der Literaturrecherche

### Psychotherapie

Im Vergleich zur ersten Version der Leitlinie gibt es in Hinblick auf Befunde zur Wirksamkeit von kognitiver Verhaltenstherapie (KVT), interpersoneller Therapie (IPT), systemischer Therapie (ST) und psychodynamischer Psychotherapie (PP) keine wesentlichen Änderungen: Für die KVT gibt es nach wie vor, auch unter Berücksichtigung aktueller Studien, am meisten Evidenz zur Wirksamkeit in Form einer Überlegenheit gegenüber Nichtbehandlung und Behandlung wie üblich. Zur IPT liegen ebenfalls Befunde zur Wirksamkeit vor, wenngleich hierzu keine neuen Studien eingeschlossen wurden. Für die ST und die PP konnten zwar neue Studien eingeschlossen werden; dennoch gibt es zu diesen Verfahren nach wie vor lediglich Hinweise zur Wirksamkeit, da keine eindeutige Überlegenheit gegenüber Nichtbehandlung und Behandlung wie üblich gezeigt werden konnte [5–12].

Für den Vergleich der verschiedenen Rahmenbedingungen (Einzel‑, Gruppen- oder Familientherapie) wurden drei neue Studien herangezogen [13–15], die jedoch keinen eindeutigen Schluss zulassen, ob eine dieser Bedingungen den anderen überlegen ist. Darüber hinaus existiert für die KVT Evidenz im onlinebasierten bzw. computergestützten Rahmen. Hierbei werden den Kindern und Jugendlichen beispielsweise Chat-Sitzungen mit einer Therapeutin/einem Therapeuten angeboten oder es werden während der Therapiesitzung Aufgaben an einem Computer bearbeitet, die von der Therapeutin/dem Therapeuten angeleitet werden. Neu aufgenommene Studien konnten zeigen, dass für eine solche online- oder computergestützte KVT mit therapeutischer Unterstützung genauso wie für eine „klassische“ KVT Befunde zur Wirksamkeit vorliegen [16–18]. Ähnliches wurde in einer Studie zur Online-PP gefunden [11], in der sowohl eigenständig Selbsthilfemodule absolviert als auch wöchentliche Chat-Sitzungen mit einer Therapeutin/einem Therapeuten durchgeführt wurden.

Im Vergleich zur Erstfassung konnten in die Revision Studien eingeschlossen werden, die Evidenz für jüngere Kinder (< 8 Jahre) enthielten. Daher wird es in der Revision getrennte Empfehlungen für die Altersgruppen 3–6 Jahre, 7–12 Jahre und 13–18 Jahre geben. Im Kindesalter gibt es vor allem Evidenz zu familienfokussierter KVT. Bei dieser Behandlungsmethode stehen die Stärkung der positiven Eltern-Kind-Interaktion im Vordergrund sowie die Stärkung der Fähigkeiten zur Stressbewältigung und die Stärkung der Emotionsregulation durch Techniken wie Problemlösetraining und Verhaltensaktivierung. In der Psychoedukation werden individuelle Faktoren erarbeitet, die zur Aufrechterhaltung der depressiven Symptomatik beim Kind beitragen und relevant für die Behandlung sind. Ein spezifischer Fokus liegt auf dem Zusammenspiel von Stimmung und zwischenmenschlichen Interaktionen sowie den Methoden zur Stressreduktion und -bewältigung in den Familien.

Für Kinder unter 8 Jahren gibt es zusätzlich noch Evidenz für die „Parent-Child-Interaction Therapy, Emotion Development“, welche spezifisch für Kleinkinder entwickelt wurde [19, 20]. Diese verhaltenstherapeutische Therapiemethode nutzt die Grundtechniken eines für Kleinkinder mit Verhaltensstörungen entwickelten Programms ([21]; Unterrichten der Eltern, gefolgt von Coaching der Eltern in der Interaktion mit dem Kind in vivo). Ziel dieser Intervention ist die Steigerung der emotionalen Kompetenz und der Emotionsregulierung des Kindes.

### Pharmakotherapie

Im Bereich der Pharmakotherapie zeigen Ergebnisse einer Übersichtsarbeit aus dem Jahr 2021 [22], dass die meisten neueren Antidepressiva die depressive Symptomatik im Vergleich zu Placebo-Medikation geringfügig reduzieren können. Diese Übersichtsarbeit ist die aktualisierte und um sieben RCTs erweiterte Version eines früheren Reviews [23], wobei die neu aufgenommenen Studien die Wirksamkeit von Duloxetin, Desvenlafaxin, Vilazodon und Vortioxetin teilweise im Vergleich mit Fluoxetin untersuchen. Während in früheren Übersichtsarbeiten und auch in der Erstfassung der Leitlinie ausschließlich Fluoxetin als Therapie der ersten Wahl gesehen wurde, gibt es nun neue Erkenntnisse, die darauf hindeuten, dass die Medikamente Sertralin, Escitalopram und Duloxetin eine ähnliche Wirksamkeit aufweisen können wie Fluoxetin. In der für die Leitlinie herangezogenen Evidenz zur Pharmakotherapie sind keine Studien vorhanden, die sich auf Kinder und Jugendliche mit einer leichten Depression und/oder Dysthymia beziehen.

Zu den klinisch hoch relevanten unerwünschten Nebenwirkungen gehört die Suizidalität. Da die Medikamente, die in der Behandlung einer Depression bei Kindern und Jugendlichen in Erwägung gezogen werden, wie z. B. Serotonin-Wiederaufnahmehemmer (SSRIs), zu einer Verstärkung von suizidalen Gedanken und einer Zunahme von suizidbedingten Handlungen führen können, sind neben einer Psychoedukation zur Psychopharmakotherapie das enge systematische Erfassen von unerwünschten Nebenwirkungen und ggf. die Anpassung der Therapiemethode notwendig. Der Großteil der Evidenz beruht hierbei auf Studien mit Jugendlichen ab 12 Jahren. Für Kinder zwischen 7 und 12 Jahren liegt eine eingeschränkte Evidenz vor, die jedoch auf die gleichen Ergebnisse hindeutet. Kinder unter 7 Jahren wurden bisher nicht in psychopharmakologische Studien eingeschlossen, sodass für diese Altersgruppe keine Evidenz vorliegt.

Neue Studien gibt es ebenfalls für das Medikament Ketamin, das vor allem in Fällen schwerer, rezidivierender Depression eingesetzt werden kann. Hierbei zeigt sich, dass Ketamin gegenüber einer Placeboinfusion überlegen sein kann [24]. Für andere Medikamente, wie z. B. trizyklische Antidepressiva, gibt es keine neue Evidenz. Die Evidenz für eine mögliche Wirkung bei Jugendlichen ist eher gering. Generell ist das Risiko für unerwünschte Nebenwirkungen, insbesondere bei trizyklischen Antidepressiva, sehr hoch.

### Kombinationsbehandlung aus Psychotherapie und Pharmakotherapie

Die Kombination eines Medikaments (z. B. SSRI) mit einer Psychotherapie zeigt im Vergleich zu den jeweiligen Monotherapien (d. h. Psychotherapie alleine oder Pharmakotherapie alleine) auch in den neu eingeschlossenen Studien keine Überlegenheit in Bezug auf einen kurz- oder mittelfristigen Therapieeffekt. Allerdings gibt es Hinweise darauf, dass der Zusatz einer kognitiven Verhaltenstherapie zu einer Medikation die Wahrscheinlichkeit eines Rückfalls bei mittelgradiger oder schwerer Depression auch längerfristig reduzieren kann [25–27].

Für die Kombinationstherapie gibt es keine Evidenz für Kinder unter 12 Jahren, hier kann also nur Evidenz für Jugendliche beschrieben werden.

### Ergänzende Therapien

Im Bereich der ergänzenden Therapien, die nicht als eigenständige Behandlung, sondern als Zusatz zu einer Psycho- oder Pharmakotherapie eingesetzt werden sollen, gibt es neue Evidenz, dass sportliche Aktivität als Zusatz zur üblichen Behandlung wirksam sein kann. Allerdings sollte hierbei beachtet werden, dass das, was in Studien unter „sportlicher Aktivität“ zusammengefasst wird, in Bezug auf Art, Frequenz, Dauer, Intensität und Modalität (mit oder ohne Anleitung einer Trainerin/eines Trainers) sehr unterschiedlich ist. Zusammenfassend kann leichte bis moderate Aktivität, wie z. B. Spazierengehen oder Fahrradfahren, wirksam sein, in einer Frequenz von ca. 3-mal wöchentlich 45–60 min, über einen Zeitraum von mindestens 6 Wochen. Hierbei kann eine Wirksamkeit sowohl alleine als auch in einer Gruppe erzielt werden [28–30].

Weitere neue Studien liegen zur Lichttherapie [31, 32] und zur Einnahme von Omega-3-Fettsäuren [33, 34] als ergänzende Therapien vor. Hierbei zeigte keine der beiden Behandlungen eine Überlegenheit gegenüber einer Placebobehandlung (Placebobehandlung mit 50 lx anstatt 2500 lx bzw. Placeboeinnahme von Omega-6-Fettsäuren anstatt Omega-3-Fettsäuren). Für andere ergänzende Therapien, wie z. B. Kunst- oder Musiktherapie, liegen keine neuen Ergebnisse vor.

Neue Evidenz wurde zur Therapie mit transkranieller Magnetstimulation (TMS) identifiziert. Ein systematisches Review über mehrere Fallserien deutet auf eine mögliche Wirkung von TMS in der Behandlung von therapieresistenter Depression hin, d. h. in den Fällen, in denen bereits mindestens eine gängige Behandlung keine oder eine nicht ausreichende Wirkung gezeigt hat [35]. Dagegen konnte TMS in einer RCT zu Jugendlichen mit therapieresistenter Depression im Vergleich zu einer Placebostimulation keine Wirkung zeigen; die Reduktion der Depressivität war in der TMS-Gruppe vergleichbar mit der in der Vergleichsgruppe, in der eine „Placebo-Spule“ eingesetzt wurde, d. h. ein Gerät, das in Art und Aussehen einer TMS-Spule gleicht, jedoch keine elektromagnetischen Impulse sendet [36]. Die Evidenz bezieht sich ausschließlich auf Jugendliche, die an einer schweren Depression leiden.

### Übergangsbereich ins Erwachsenenalter

Es wurden keine randomisierten kontrollierten Interventionsstudien identifiziert, die eine spezifische Behandlung für den Transitionsbereich oder ein spezifisches Setting, wie z. B. eine Transitionsstation, evaluierten. Eine nicht-vergleichende Interventionsstudie, welche eine Behandlung mit dem Fokus auf transitionsspezifische Themen untersuchte, konnte zeigen, dass eine solche Intervention auf gute Akzeptanz stoßen kann, weiterempfohlen werden würde und die Aussichten der Betroffenen auf die Zukunft verbessern kann [37].

## Nächste Schritte

Nach der Übereinkunft des Steuerungsgremiums in Hinblick auf die formulierten evidenz- und konsensbasierten Empfehlungen findet die Abstimmung über die Empfehlungen im Konsentierungsgremium statt. Dazu finden sich Vertreter*innen aus relevanten Fachverbänden in einem Delphi-Verfahren zusammen, in welchem eine Online-Abstimmung getätigt wird. Hierbei können die Fachvertreter*innen ihre Zustimmung zu den vorgegebenen Empfehlungen geben oder ihre Ablehnung begründen und Verbesserungsvorschläge einreichen. Empfehlungen, zu denen bei Abschluss des Verfahrens eine mindestens 75-prozentige Zustimmung vorliegt, werden in der Revision der Leitlinie verabschiedet. Nach Konsentierung der Empfehlungen wird die Revision bei der Arbeitsgemeinschaft der Wissenschaftlichen Medizinischen Fachgesellschaften e. V. (AWMF) eingereicht und veröffentlicht. Im Verlauf sind, wie schon nach der Erstfassung, weitere Evaluationen der Leitlinie geplant.

## Fazit

Die Revision der S3-Leitlinie zur Behandlung depressiver Störungen im Kindes- und Jugendalter wird mehrere relevante Neuerungen aufweisen. Zum einen wurden neue Schlüsselfragen in Bezug auf ergänzende Therapien und den Übergangsbereich vom Jugend- ins Erwachsenenalter und damit verbundene neue Empfehlungen formuliert. Zum anderen gibt es neuere Studien zu den Behandlungsmethoden, sodass mit Änderungen an den Empfehlungen zu diesen Bereichen zu rechnen ist. Die Veröffentlichung der Revision der Leitlinie ist für Ende 2023 geplant. Ohne die Entscheidung des Konsentierungsgremiums vorweggreifen zu wollen, könnten beispielsweise neben Fluoxetin weitere Medikamente wie Escitalpram, Duloxetion und Sertralin als Medikamente der ersten Wahl empfohlen werden. Außerdem wird es erstmals therapeutische Empfehlungen für jüngere Kinder (< 8 Jahre) geben. Zu dieser Altersgruppe liegt Evidenz für den Einsatz von familienfokussierter KVT vor.
